# Hardware Implementation of Improved Oriented FAST and Rotated BRIEF-Simultaneous Localization and Mapping Version 2

**DOI:** 10.3390/s25206404

**Published:** 2025-10-17

**Authors:** Ji-Long He, Ying-Hua Chen, Wenny Ramadha Putri, Chung-I. Huang, Ming-Hsiang Su, Kuo-Chen Li, Jian-Hong Wang, Shih-Lun Chen, Yung-Hui Li, Jia-Ching Wang

**Affiliations:** 1 School of Computer Science and Technology, Shandong University of Technology, Zibo 255000, China; 23505020692@stumail.sdut.edu.cn (J.-L.H.); jhwang@sdut.edu.cn (J.-H.W.); 2 Department of Computer Science and Information Engineering, National Central University, Taoyuan City 320317, Taiwan; alyson@g.ncu.edu.tw (Y.-H.C.); wenny@g.ncu.edu.tw (W.R.P.); jcw@csie.ncu.edu.tw (J.-C.W.); 3 National Center for High-Performance Computing, Hsinchu 300092, Taiwan; 1203033@narlabs.org.tw; 4 Department of Data Science, Soochow University, Taipei 111002, Taiwan; 5 Department of Information Management, Chung Yuan Christian University, Taoyuan City 320314, Taiwan; 6 Department of Electronic Engineering, Chung Yuan Christian University, Taoyuan City 320314, Taiwan; chrischen@cycu.edu.tw; 7 AI Research Center, Hon Hai Research Institute, New Taipei City 207236, Taiwan; yunghui.li@foxconn.com

**Keywords:** spatial scene construction, machine vision, SLAM, ORB-SLAM2, Raspberry Pi 3

## Abstract

The field of autonomous driving has seen continuous advances, yet achieving higher levels of automation in real-world applications remains challenging. A critical requirement for autonomous navigation is accurate map construction, particularly in novel and unstructured environments. In recent years, Simultaneous Localization and Mapping (SLAM) has evolved to support diverse sensor modalities, with some implementations incorporating machine learning to improve performance. However, these approaches often demand substantial computational resources. The key challenge lies in achieving efficiency within resource-constrained environments while minimizing errors that could degrade downstream tasks. This paper presents an enhanced ORB-SLAM2 (Oriented FAST and Rotated BRIEF Simultaneous Localization and Mapping, version 2) algorithm implemented on a Raspberry Pi 3 (ARM A53 CPU) to improve mapping performance under limited computational resources. ORB-SLAM2 comprises four main stages: Tracking, Local Mapping, Loop Closing, and Full Bundle Adjustment (BA). The proposed improvements include employing a more efficient feature descriptor to increase stereo feature-matching rates and optimizing loop-closing parameters to reduce accumulated errors. Experimental results demonstrate that the proposed system achieves notable improvements on the Raspberry Pi 3 platform. For monocular SLAM, RMSE is reduced by 18.11%, mean error by 22.97%, median error by 29.41%, and maximum error by 17.18%. For stereo SLAM, RMSE decreases by 0.30% and mean error by 0.38%. Furthermore, the ROS topic frequency stabilizes at 10 Hz, with quad-core CPU utilization averaging approximately 90%. These results indicate that the system satisfies real-time requirements while maintaining a balanced trade-off between accuracy and computational efficiency under resource constraints.

## 1. Introduction

As artificial intelligence technology has advanced, various fields have increasingly focused on artificial intelligence (AI) research, developing machine learning and deep learning techniques that are now applied in real-world scenarios. Among the many topics in deep learning, visual image processing is one of the most extensively explored areas. Researchers often use deep neural network models to investigate applications such as image classification, segmentation, and recognition, which have practical uses in every-day life, including action recognition [1], medical image segmentation [2], iris image seg-mentation [3], calcaneus fracture detection [4], animal species classification, speech recognition [5], guitar playing technique recognition [6], and sound recognition [7].

In recent years, self-driving cars have gradually become widely discussed. From various works of television and film, one can see people’s fantasies and expectations for the future of autonomous vehicles. Returning to the real world, the realization of autonomous driving technology relies on sensory systems such as cameras, LiDAR, radar, positioning systems, path planning systems, and so on. Combined with artificial intelligence and algorithms, it can further interact with humans, for example, through voice intelligent assistants. The level of automation of self-driving cars is classified into 0 to 5 levels, as defined by the National Highway Traffic Safety Administration [8].

Self-driving cars play an increasingly vital role in modern society. To achieve efficient and accurate navigation and operation, self-driving cars require the integration of various advanced technologies, including machine vision, motor control, the Robot Operating System (ROS) [9], and Simultaneous Localization and Mapping (SLAM) [10,11,12].

Machine vision is a key component of the self-driving cars’ navigation system, enabling self-driving cars to perceive the surrounding environment and identify obstacles, path markers, and target objects. By equipping cameras and other sensors, self-driving cars can capture rich visual information, which, after processing through image processing and pattern recognition algorithms, is transformed into an environmental model that self-driving vehicles can comprehend.

Motor control is at the heart of self-driving cars’ motion control, involving precise speed and direction control to ensure that self-driving cars travel smoothly along predetermined paths. Motor control systems typically include drivers, encoders, and control algorithms that work together to achieve precise control over the movement of self-driving cars.

Robot Operating System (ROS), an open-source robotics software platform [9], provides robust support for developing self-driving cars. It offers a set of standardized communication protocols and tools that enable seamless integration of various hardware and software components.

SLAM [10] is another crucial technology in the self-driving cars’ navigation system, allowing self-driving cars to perform autonomous localization and map building in unknown environments. By integrating data from sensors such as Light Detection and Ranging (LiDAR), cameras, and Inertial Measurement Units (IMUs), SLAM systems can create environmental maps in real-time and simultaneously estimate the position of the self-driving car within the map.

In these systems, stable and accurate localization mapping is crucial. Any anomalies or errors in this aspect can lead to cumulative inaccuracies in subsequent applications, resulting in an imperfect overall system. In practical research and project execution, there may not always be sufficient hardware resources to handle extensive image processing. Many consumer electronic products opt for Advanced Reduced Instruction Set Computer Machine (ARM) architecture systems, selecting corresponding chips based on the project’s requirements. Therefore, this paper will use the commonly available Raspberry Pi 3 (ARM A53) as an example to implement improvements to ORB-SLAM2. The choice of ORB-SLAM2 is due to its support for three camera modes: monocular, stereo, and RGB-D. In monocular and stereo modes, feature points represent the SLAM map style, reducing the need for extensive image processing steps such as image stitching, compared to RGB-D.

Since most product developments are based on ARM architecture, this paper utilizes the commonly available Raspberry Pi 3 (ARM A53) as an example to implement Visual-SLAM applications on the platform. Standard Visual-SLAM systems include RTAB-MAP [13], RGB-D-SLAM [14], and ORB-SLAM [10,15,16,17] series.

Although ORB-SLAM3 [17] was introduced with enhancements including support for image odometry and a wider range of camera inputs, these improvements also entail increased computational demands, especially on platforms with limited hardware resources. Therefore, this paper builds upon ORB-SLAM2 [15] as a foundation, focusing solely on sparse mapping using feature points from monocular and stereo inputs. The aim is to make ORB-SLAM2 [15] more suitable for operation on platforms with constrained hardware capabilities. The discussion begins with exploring feature point matching to assess whether employing new matching techniques enhances quantitative accuracy and correctness. Additionally, improvements will be made to the four components of ORB-SLAM2 using alternative methods.

Due to constraints in hardware resources, this paper only investigates the use of monocular and stereo vision, starting with the fundamentals of stereo vision. There will be no further exploration into RGB-D image stitching.

Numerous open-source SLAM variants are available and classified according to the sensors they utilize. They can be broadly categorized into three main types: “RGB-D (stereo or multi-camera)”, “monocular”, and “Lidar”, as illustrated in Table 1.

Monocular SLAM systems reconstruct three-dimensional information from the two-dimensional images captured by a single camera. MonoSLAM [18] enables real-time 3D trajectory recovery of rapidly moving cameras. At the same time, Parallel Tracking and Mapping (PTAM) [19] provides a camera tracking system for augmented reality without the need for additional markers or devices, demonstrating efficient performance under resource-constrained conditions. LSD-SLAM [21,22] addresses feature matching problems in texture-poor environments by directly processing image intensities.

Depth-sensing SLAM systems utilize color images and depth information obtained from RGB-D cameras for 3D modeling and robot localization, which is particularly suitable for indoor environments and effective in handling dynamic scenes. RGB-D SLAM [14] technology, by combining these two types of information, can perform real-time 3D modeling of unknown environments and achieve self-localization of robots. Dense Tracking and Mapping [24] offers precise camera tracking and scene reconstruction by densely tracking every pixel.

Multi-sensor SLAM systems enhance performance by integrating data from various sensors. ORB-SLAM [10,15,16,17,20] supports multiple camera types, offering a universal SLAM solution. Semi-Direct Visual Odometry [23] provides accurate visual odometry for micro aerial vehicles in GPS-denied environments. RTAB-Map [13] and Elastic Fusion [27] achieve precise real-time localization and dense mapping by supporting a variety of sensors. Hector SLAM [28] is specifically designed for search and rescue robots, enhancing SLAM capabilities in complex environments. These systems showcase the high adaptability and potential of SLAM in diverse settings.

The smartphone-based ORB-SLAM and Pedestrian Dead Reckoning (PDR) Inertial Sensor Fusion System [29] accomplishes the backend data fusion between the monocular camera and PDR inertial sensors (accelerometers, gyroscopes, magnetometers) via Kalman Filtering, which effectively corrects the cumulative errors and drift issues of a single system; meanwhile, DOG-SLAM [30], an RGB-D SLAM system optimized for dynamic environments, achieves accurate segmentation of dynamic regions and sufficient retention of static feature points by adopting the pseudo-semantic segmentation strategy based on Gaussian Mixture Model (GMM) and dynamic pre-removal strategy. It also introduces Feature Booster to generate ORB-Boost descriptors, enhancing the matching robustness of static feature points.

The types of sensors are also exemplified in Table 2 [31,32,33].

The advantage of RGB-D cameras lies in their ability to utilize various algorithms and triangulation techniques to acquire 3D data. RGB-D cameras capture the scene with an infrared depth sensor to calculate the depth of each pixel, while the RGB camera captures the color image of the scene; the depth data is then correlated with the color image to obtain an RGB image with per-pixel depth information This capability to combine depth and color information makes RGB-D cameras perform well in 3D modeling, object recognition, and scene understanding, especially suitable for indoor environment mapping and robot navigation applications [31].

Lidar cameras can provide accurate three-dimensional spatial information. Compared with other sensors such as infrared, radar, and ultrasonic, laser scanners are not limited by lighting conditions when detecting targets and can provide more precise measurement results [33].

ORB-SLAM [10,15,16,17,20] was published and open-sourced, but the first-generation version did not support inputs from multiple sensors, only enabling SLAM with monocular cameras. This paper adopts the open-sourced ORB-SLAM2 [15] as its foundation. However, ORB-SLAM2 still had shortcomings in mapping rotations, prompting the same team to release ORB-SLAM3 [17], which utilized IMU integration to enhance mapping stability.

Despite the advancements in ORB-SLAM3, its higher computational and resource requirements make it less accessible for some applications where hardware constraints are a concern. As a result, ORB-SLAM2 is often the preferred choice for its balance between performance and efficiency. In the context of this paper, ORB-SLAM2 has been selected as the foundation for spatial modeling due to its proven reliability and the relative ease of implementation, especially when the computational resources are limited.

**Figure 1:**
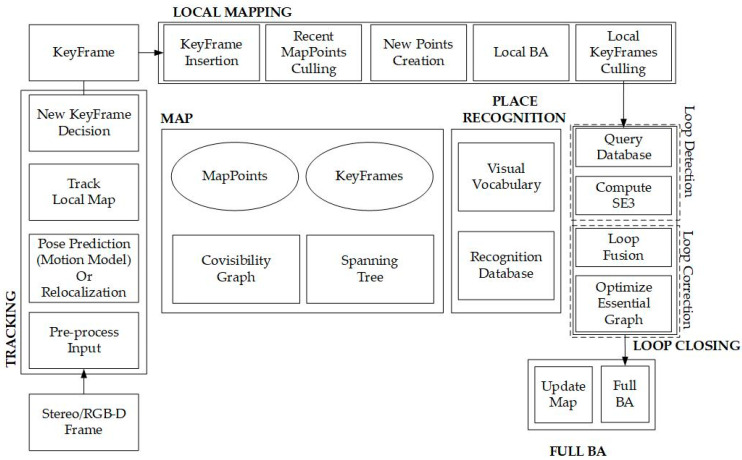
Operational Workflow of ORB-SLAM2.

The overall operational workflow can be divided into four main parts: “TRACKING,” “LOCAL MAPPING,” “LOOP CLOSING,” and “FULL BA (Bundle Adjustment).” The “FULL BA” is executed only after the confirmation of the “LOOP CLOSING” phase completion. The operational workflow of ORB-SLAM2 is shown in Figure 1 [15].

In the Operational workflow of ORB-SLAM2:

- TRACKING: Matches local map features by searching and pre-processing (Extract ORB) in this phase.
- LOCAL MAPPING: Manages and optimizes local maps through local bundle adjustment (BA).
- LOOP CLOSING: Detects large loops, optimizes pose, and corrects drift errors.
- FULL BA: Computes the optimal structure and motion results for the entire system after pose optimization.

The primary contribution of this work lies in enhancing the ORB-SLAM2 algorithm for resource-constrained environments, with a focus on the Raspberry Pi 3 Model B. Rather than improving the Boosted Efficient Local Descriptor (BELID) itself, the novelty of this work stems from the targeted integration of BELID with ORB features and the collaborative optimization of back-end loop-closing weight parameters. This design is tailored to the computational limits of the ARM A53 CPU, ensuring that the system achieves a practical balance between accuracy and real-time performance on embedded platforms. Quantitative evaluations demonstrate that for monocular SLAM, RMSE, mean, and median errors are reduced by 18.11%, 22.97%, and 29.41%, respectively. In comparison, the maximum error decreases by 17.18%, significantly enhancing trajectory estimation and reliability under challenging conditions. Although stereo SLAM improvements are relatively minor, with RMSE and mean error reduced by 0.30% and 0.38% and a slight 1.49% increase in median error, these refinements still contribute to greater overall stability and efficiency. The proposed adaptations offer a feasible pathway for deploying advanced SLAM algorithms on low-power embedded devices, underscoring their applicability to real-world scenarios such as autonomous navigation and mobile robotics.

## 2. Research Method

### 2.1. Hardware Architecture

**Figure 2:**
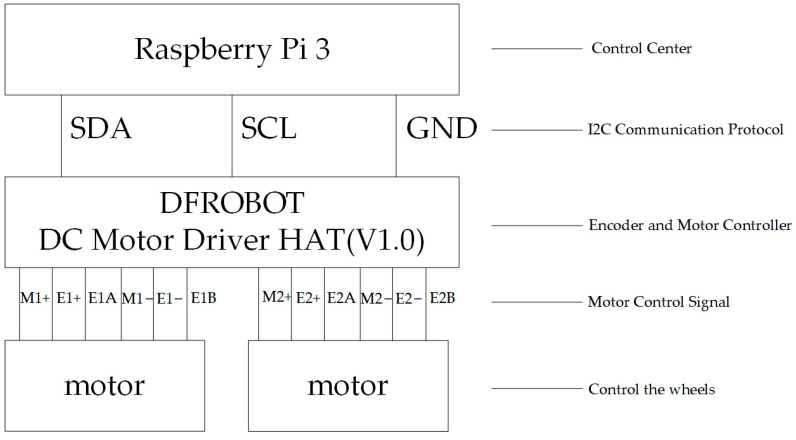
Connection of the motor and mini encoder.

**Figure 3:**
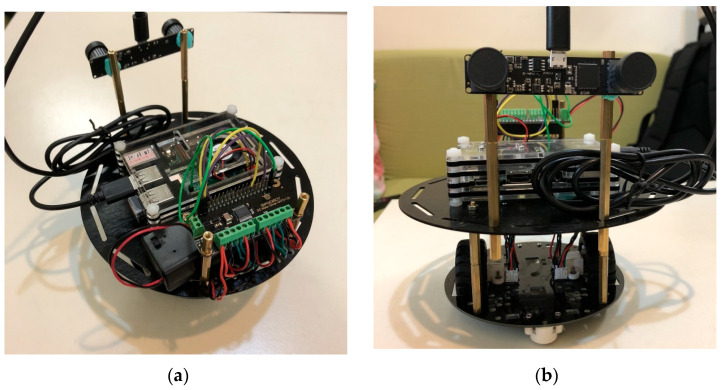
Physical Assembly Diagram: (**a**) Vertical View. (**b**) Front View.

Because the Raspberry Pi 3 processes images relatively slowly, mini encoders were added to control the stability and speed of rotation in the motor control section, ensuring the smooth operation of the overall system. They are connected via the DFR0592 expansion board, as shown in Figure 2 [34]. The physical assembly diagram is shown in Figure 3.

**Figure 4:**
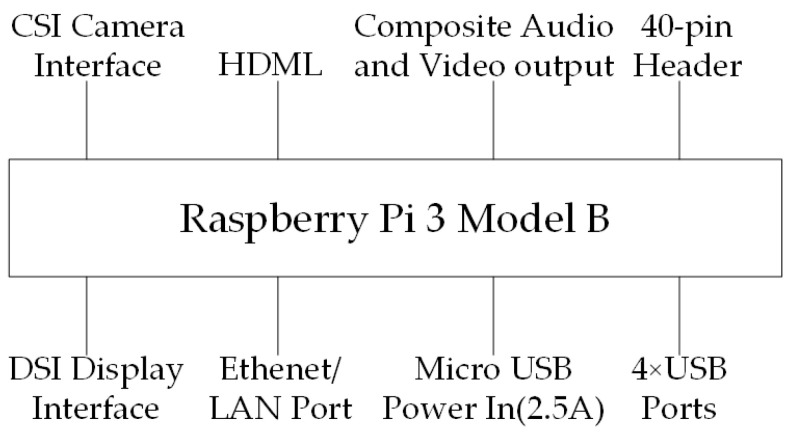
Raspberry Pi 3.

This paper uses the Raspberry Pi 3 Model B as the platform base, as shown in Figure 4 [35]. The Raspberry Pi 3 Model B is a single-board computer driven by the Broadcom BCM2837 chipset, featuring a 1.2 GHz 64-bit quad-core ARM Cortex-A53 processor. It comes with built-in 802.11 b/g/n wireless LAN and Bluetooth 4.1 (including Classic and Low Energy BLE) and a dual-core Video core IV^®^ multimedia coprocessor. Its interfaces include a Micro USB power connector (supporting 2.5A power supply), a 10/100 Ethernet port, an HDMI video/audio connector, an RCA video/audio connector, four USB 2.0 ports, 40 GPIO pins, a DSI display connector, and a microSD card slot. Additionally, it is equipped with an on-board antenna for wireless connectivity.

A special USB Stereo Camera is employed for the camera section, which outputs two combined images at a resolution of 640 × 240 pixels. The paper presents a custom program developed under the ROS architecture to split the image into two 320 × 240 pixel left and right views. The left image is then utilized as the input for monocular camera experiments. The camera used in this study is from Shenzhen RERVISION Technology Co., Ltd., Shenzhen city, China. The camera parameters are shown in Table 3 [36]. 

Due to the sensitivity of ORB-SLAM2 mapping quality to movement speed, a mini encoder was implemented to control the movement speed on the actual robot platform. The DFRobot MiniQ encoder was utilized for this purpose, as shown in Table 4 [37].

**Figure 5:**
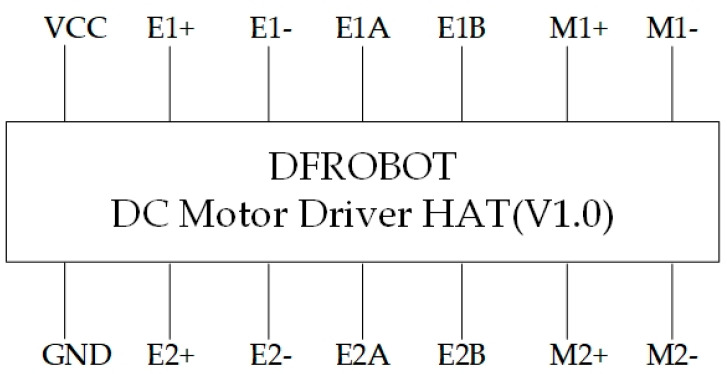
Encoder and Motor Controller Utilized.

The Raspberry Pi 3 expansion board DFR0592 was employed to ensure smooth operation of the entire system, as depicted in Figure 5 [38]. This is a Raspberry Pi DC motor driver board with an on-board encoder interface, which can drive a 2-way DC motor and a DC motor with encoder [38]. By utilizing the I2C interface, the expansion board enables the vehicle to move at a slow speed of 5–10 rpm.

### 2.2. Software Architecture

**Figure 6:**
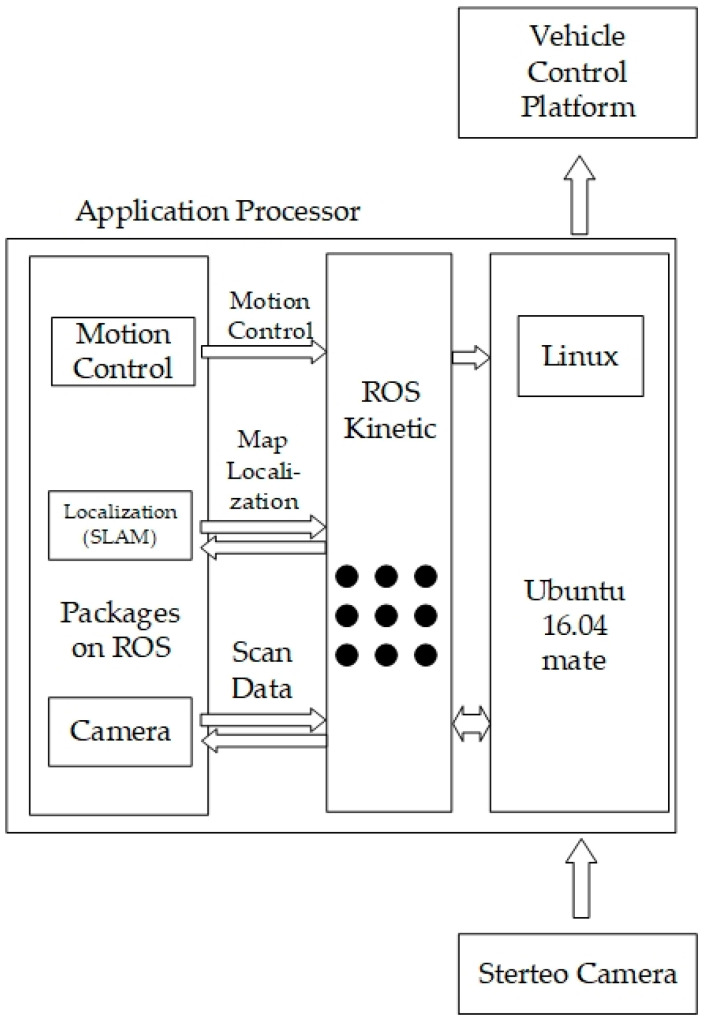
System architecture diagram.

**Figure 7:**
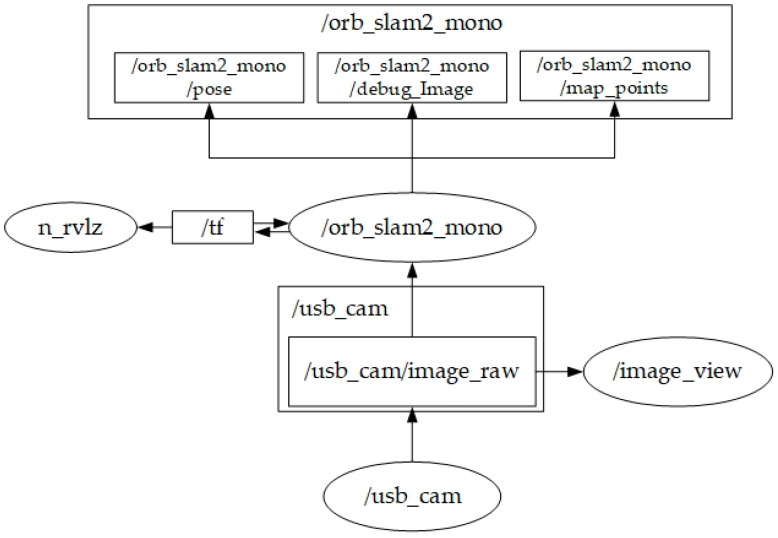
ROS node relationships for Mono Camera ORB SLAM 2.

**Figure 8:**
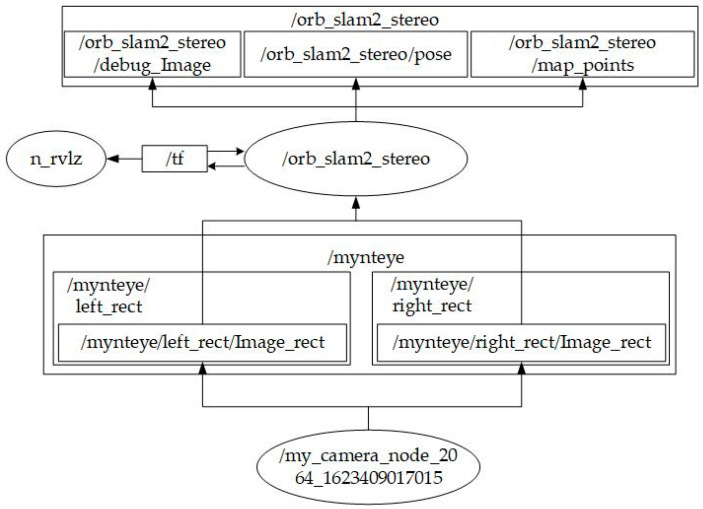
ROS node relationships for Stereo camera ORB SLAM 2.

Built upon Ubuntu 16.04 Mate on the Raspberry Pi 3, the system utilizes ROS Kinetic as the overarching middleware, governing all inputs (monocular or stereo cameras) and outputs (motor control). The corresponding system architecture is depicted in Figure 6, Figure 7 and Figure 8 [9]. They illustrate the ROS node relationships in monocular and stereo scenarios, respectively.

The ROS topics used here are around 10 Hz, with CPU usage averaging approximately 90% across four cores. The CPU core usage may vary depending on different experimental scenarios.

### 2.3. Image Descriptors

Image descriptors are fundamental tools in computer vision, designed to capture and represent salient image features. They generate compact representations for detected structures, enabling robust feature matching across different views. An adequate descriptor must be discriminative, computationally efficient, and resilient to changes in viewpoint, illumination, and partial occlusions. Such properties make descriptors indispensable in tasks such as 3D reconstruction, SLAM (Simultaneous Localization and Mapping), image retrieval, object recognition, and pose estimation. BRIEF (Binary Robust Independent Elementary Features) [39] is a widely used binary descriptor that encodes local image patches by comparing the intensity of pixel pairs. Each comparison yields a binary result, and the concatenation of these results forms the descriptor. BRIEF is known for its speed and robustness to noise and illumination variations, which makes it suitable for real-time applications. In ORB-SLAM2, the ORB descriptor [15] combines FAST corner detection with BRIEF descriptors, extending them with rotational invariance while preserving computational efficiency.

The Boosted Efficient Local Image Descriptor (BELID) [40] was introduced to improve real-time image matching on resource-constrained platforms. BELID employs the AdaBoost algorithm with an improved weak-learner training scheme, integrating binary encoding to minimize computational overhead while maintaining competitive accuracy. It achieves accuracy comparable to SIFT while operating with a runtime similar to ORB, making it particularly suitable for embedded systems. Formally, BELID uses a training set ${\left\{(x_{i}\;,y_{i}\;,{\;l}_{i})\right\}}_{i=1}^{N}$, where $x_{i}\;,\;y_{i}\;\in X$ are image patches, and $l_{i}\in \{-1,1\}$ indicates whether the two patches correspond to the same feature structure $\left(l_{i}=1\right)$ or not ($l_{i}=-1)$. The BoostedSCC framework trains weak learners $h_{k}(\cdot )$ to minimize the following exponential loss [40]:

(1) $$L_{BSCC}=\sum_{i=1}^{N}exp(-l_{i}\sum_{k=1}^{K}a_{k}h_{k}(x_{i})h_{k}(y_{i}))$$

where $h_{k}\left(x;f,T\right)$ is the *k*-th weak learner defined by a feature extraction function f and a threshold T:

(2) $$h(x;f,T)\;=\;\left\{\begin{array}{c} +1\;if\;f(x)\leq T\\ -1\;if\;f(x)>T \end{array}\right.$$

**Figure 9:**
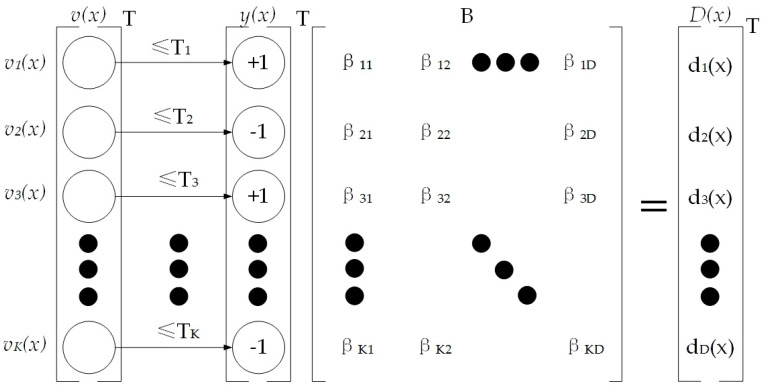
Descriptor extraction process of BELID. A set of weak learners evaluates local image patches by comparing regional intensity differences against learned thresholds $T_{k}$, producing binary responses {−1,1}. These responses are weighted by the learned coefficients $\beta_{kd}$ within the BoostedSCC framework, and aggregated into a compact binary vector D(x), which serves as the final descriptor.

As shown in Figure 9, during descriptor construction, BELID selects a local image patch and samples *K* pairs of square regions. For each pair, the mean grayscale values are computed, and their difference is obtained efficiently using integral images. Each difference serves as the response of a weak learner, which is then binarized according to the learned threshold. The BoostedSCC algorithm optimizes the weights of these weak learners, emphasizing the most discriminative features. The final descriptor D(x) is a compact binary vector formed by aggregating the responses of all weak learners. By combining discriminative power, binary encoding, and computational efficiency, BELID offers a practical balance between accuracy and speed. These characteristics make it particularly advantageous for adapting ORB-SLAM2 to embedded platforms such as the Raspberry Pi 3, where computational resources are severely limited.

## 3. Experimental Architecture and Steps

### 3.1. EuRoC MAV Dataset

The EuRoC MAV dataset [41] is a valuable resource designed and collected to evaluate visual-inertial localization algorithms. It includes synchronized stereo images, IMU measurements, and accurate ground truth data captured on a micro aerial vehicle (MAV). The dataset is divided into two batches; the first batch, collected in an industrial environment, provides location information with millimeter-level accuracy, while the second batch focuses on the precise reconstruction of 3D environments, recorded in an indoor environment equipped with a motion capture system. In total, there are 11 datasets covering a variety of scenarios from slow to dynamic flight, including motion blur and poor lighting conditions, offering researchers a comprehensive opportunity to test and evaluate algorithms. The dataset includes raw sensor measurements, spatially and temporally aligned sensor data, extrinsic and intrinsic calibration data, and custom calibrated datasets. These rich resources are not only suitable for the evaluation of visual-inertial localization algorithms but also for the assessment of appearance-based localization, monocular visual odometry, SLAM, and online 3D reconstruction algorithms, providing strong support for research on micro aerial vehicles in urban streets, industrial, and indoor environments.

The hardware setup includes the following equipment list:

- Aircraft Platform: AscTec Firefly.
- Stereo VIO Cameras: Global shutter, monochrome, operating at a frequency of 20 Hz, with hardware (HW) synchronization between the camera and IMU. The stereo camera model is MT9V034, and the IMU model is ADIS16448.
- VICON: Reflective markers used in conjunction with the VICON motion capture system.
- LEICA: Sensing prism associated with the laser tracking system.

### 3.2. Experimental Scene Architecture

**Figure 10:**
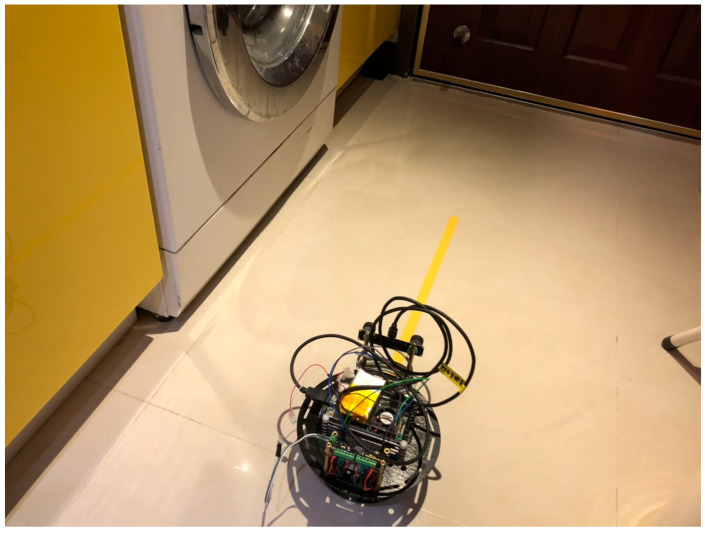
Schematic Diagram of Real Scene.

Regarding the experimental scene, we utilize real-world environments as depicted in Figure 10, along with the EuRoC MAV Dataset MH_01_easy scene [41]. By employing the same scene from the dataset alongside absolute IMU values as a reference, we aim to assess whether the refined algorithms offer further improvements in accuracy.

## 4. Experimental Results

### 4.1. Real Scene Data

Test Scenario 1 depicts the vehicle advancing within a small room (yellow lines on the ground denote a distance of 60 cm), as shown in Figure 10. ORB+BELID preprocessing is applied to the input from the stereo camera. During the experiment, it can be observed that the vehicle starts marking feature points after moving forward approximately 30 cm, and drifts while calculating the forward position (position x = 77). The forward distance error is approximately 2 cm (measured with a ruler). The instability in calculating feature points may be attributed to the dim lighting conditions in the room.

**Figure 11:**
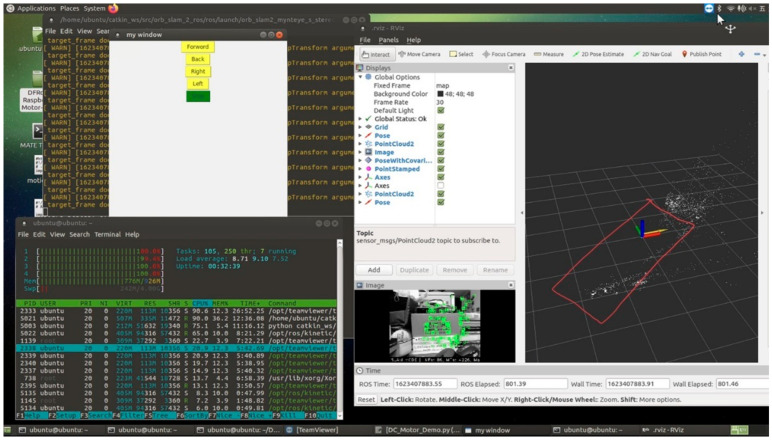
Experiment scenario 2 (ORB+BELID).

Test Scenario 2 involved mapping the entire room by rotating the vehicle. However, due to suboptimal parameter adjustments, only the initial and final loop closures correctly captured the position. It is also possible that the time taken for computation did not match the movement speed. Subsequently, attempting to wait for a period after each rotation resulted in successfully mapping the entire room. The red lines in Figure 11 represent a schematic diagram of the actual experimental room, among which the red, green, and blue arrows represent the spatial rectangular coordinate system, and the yellow arrows represent the movement direction of the vehicle. From this diagram, it can be observed that sparse mapping cannot be established within a scenario where the distance from the camera is within 40 cm of the focal length. The method used here is ORB+BELID, with the CPU performance utilized being approximately 98% of four cores.

**Figure 12:**
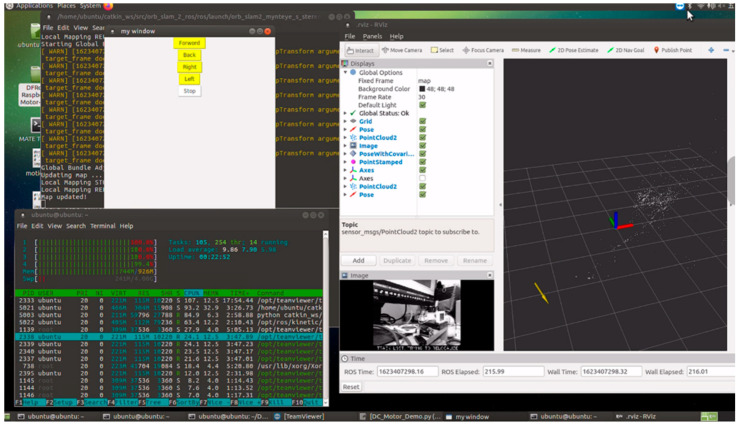
Experiment scenario 3 (ORB+BRIEF).

Experiment Scenario 3: Comparing the performance with the original ORB-SLAM2 (ORB+BRIEF), as shown in Figure 12, the CPU performance is utilized at approximately 97% of four cores, similar to the performance after improvement. In the figure, the yellow pose indicates ongoing rotational positioning.

### 4.2. Experimental Performance Comparison

In SLAM evaluation, Root Mean Squared Error (RMSE) is commonly employed to quantify trajectory accuracy, representing the deviation between the estimated trajectory and the ground truth. The mean error reflects the overall performance across multiple experiments, while the median provides a robust estimate less affected by outliers. Standard deviation indicates the consistency of results, whereas the minimum and maximum errors capture the system’s best-case and worst-case performance, respectively. The baseline results of the original ORB-SLAM2 (using ORB+BRIEF) are presented in Table 5. These results provide a reference point for subsequent optimizations.

Subsequently, when replacing BRIEF with BELID (Table 6), only marginal changes in accuracy were observed, and CPU utilization remained comparable to the original method. This confirms that the adoption of BELID alone does not yield substantial improvements on the Raspberry Pi platform. Detailed comparisons are illustrated in Figure 11 and Figure 12.

To further investigate parameter adjustments under the computational limits of the Raspberry Pi 3, the feature dimension was increased from the default 256 to 512 (Table 7). While this modification resulted in slight improvements in the stereo configuration, it did not lead to substantial accuracy gains in monocular SLAM.

Recognizing that improvements in feature matching may result from multiple factors, and following the approach suggested in [41], additional experiments were conducted by doubling the number of detected feature points while keeping the feature dimension fixed at 256 (Table 8). This adjustment led to a substantial improvement in monocular SLAM accuracy, while only marginal gains were observed in the stereo configuration.

Finally, motivated by prior work on enhanced loop-closure strategies [42], the back-end loop-closing module was further optimized by assigning higher weights to matched feature frames with stronger similarity scores. This parameter adjustment effectively reduced trajectory error values under constrained computational resources, as shown in Table 9.

Overall, these results demonstrate that while BELID alone does not significantly enhance accuracy, systematic parameter adjustments—particularly in feature point density and loop-closing weight optimization—enable a balanced improvement in accuracy and stability. These findings highlight the importance of tailoring algorithmic configurations to the computational limitations of embedded platforms.

**Figure 13:**
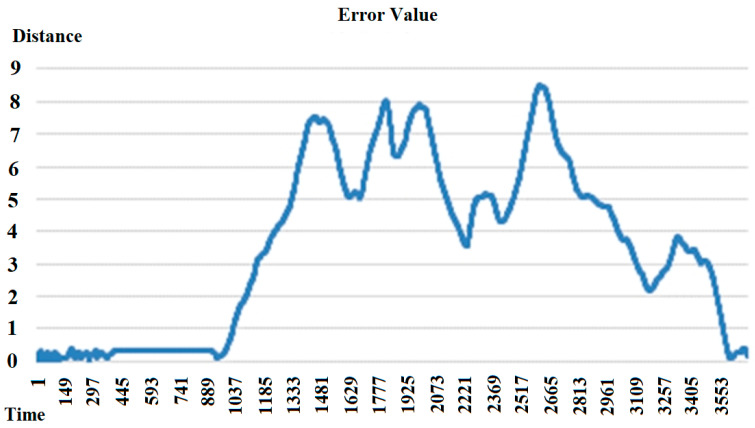
Relationship between distance and error values.

Due to the behavior of the dataset, which involves circling the experimental room from an origin point and returning to the same point. From Figure 13, with time on the horizontal axis and error values on the vertical axis, it is observed that as the distance from the origin point increases, the accumulated error values also increase.

Based on the above results, the improved ORB-SLAM2 algorithm shows only a minor enhancement in stereo camera performance. The RMSE decreased from 0.2323 m to 0.2316 m (0.30% reduction), and the mean error dropped by 0.38%. However, the median error increased slightly by 1.49%, and the maximum error increased by 0.99%. Notably, the minimum error significantly rose from 0.0068 m to 0.1275 m, suggesting increased variability in the best-case scenario. These findings indicate that, for stereo SLAM, the proposed optimization strategy produces only marginal gains while introducing inevitable trade-offs in error distribution. The improvements to the monocular camera are more substantial. RMSE decreased from 3.5948 m to 2.9437 m, an 18.11% reduction, and the mean error dropped by 22.97%. The median error saw a 29.41% decrease, demonstrating better overall accuracy. The maximum error was also reduced by 17.18%, indicating better worst-case performance. However, the standard deviation increased by 23.01%, implying higher variability in error distribution. These results suggest that the proposed optimizations are particularly effective for monocular SLAM, but they also highlight the inherent trade-offs between accuracy gains and stability in error dispersion.

These results demonstrate that parameter tuning guided by computational constraints and algorithmic logic can yield meaningful improvements in embedded SLAM performance, especially under monocular settings. Nevertheless, the increased variability and the limited stereo performance gains indicate that further investigation is required to generalize the optimization principles. Due to constraints in the research workforce and project timeline, this study could not exhaustively explore alternative optimization strategies. Future work will systematically evaluate different parameter optimization principles (e.g., computation-only, accuracy-only, and hybrid schemes), apply statistical validation across diverse datasets, and explore theoretical formulations to strengthen the generalizability of these guidelines.

## 5. Conclusions and Future Prospects

The experiments conducted in this study show that under limited resource conditions, replacing the feature point descriptors in the front end and adjusting the parameters of the LOOP CLOSING in the back end can improve accuracy. At the same time, when attempting within the range that the Raspberry Pi 3 can handle, increasing the feature dimension from the default of 256 to 512, it can be seen from the results of the stereo part that there is an improvement. In addition, we tried to double the number of feature points detected in a single image. We found a significant improvement in accuracy observed in monocular photos, and a slight improvement in the stereo part. In this paper, we have successfully implemented an improved version of the ORB-SLAM2 algorithm, which runs on the Raspberry Pi 3 Model B platform. By adopting a more efficient descriptor, we have significantly improved the matching rate of stereo matching feature points, enhancing the algorithm’s robustness. In addition, we have made detailed adjustments to the parameters in the loop detection to correct the cumulative errors that may occur in the map-building process. Through precise initialization in the front end and continuous optimization in the back end, we have ensured that even on the CPU ARM A53 architecture with limited resources, satisfactory positioning accuracy can be achieved. These improvements enhance the algorithm’s performance on resource-constrained devices and provide proof of feasibility for deploying advanced SLAM systems on similar hardware platforms.

Despite the notable improvements achieved on the Raspberry Pi 3, there remains substantial potential for further optimization. Future work will focus on enhancing the algorithm’s overall performance, including developing more efficient feature extraction techniques, innovative loop-closure detection strategies, and optimized multi-sensor data fusion. Advanced machine learning and deep learning methods will be explored to improve feature point recognition and matching capabilities. At the same time, more sophisticated graph optimization algorithms will be investigated to reduce cumulative errors and enhance long-term localization stability. Given the hardware constraints of the Raspberry Pi 3, future research will also examine effective parallel processing and hardware acceleration strategies to maximize computational efficiency. These efforts aim to improve algorithm execution speed and extend applicability to complex scenarios. Additionally, a wider range of test conditions—including varying lighting environments, indoor/outdoor settings, and diverse scene complexities—will be incorporated to evaluate robustness comprehensively. Comparisons with other SLAM approaches, such as ORB-SLAM2, LSD-SLAM, and RTAB-Map, will be conducted to validate performance further. Finally, more detailed analyses of computational resource usage and accuracy, supplemented with appropriate statistical tests, will be included to provide precise and reliable performance metrics. These ongoing efforts aim to advance visual SLAM technologies and facilitate deployment in broader application domains, including autonomous driving, robotic navigation, and uncrewed aerial vehicle operations.

## Figures and Tables

**Table 1:** Standard Introductions to SLAM.

SLAM Algorithm	Types of Sensors
RGB-DSLAM [14]	RGB-D
GMapping [16]	Lidar
MonoSLAM [18]	Monocular
Parallel Tracking and Mapping [19]	Monocular
ORB-SLAM(2)(3) [10,15,16,17,20]	Monocular/stereo/RGB-D
LSD-SLAM [21,22]	Monocular
Semi-Direct Visual Odometry [23]	Monocular
Dense Tracking and Mapping [24]	RGB-D
Depth From Videos [25]	Monocular
Direct Sparse Odometry [26]	Monocular
RTAB-MAP [13]	Stereo/RGB-D/Lidar
Elastic Fusion [27]	RGB-D
Hector SLAM [28]	Lidar
ORB-SLAM and PDR Inertial Sensors Fusion [29]	Monocular
DOG-SLAM [30]	RGB-D

**Table 2:** Sensor Specification.

Parameter	Value		
Model	Asus XtionPro Live	Basler acA2000-50gc	Hokuyo UTM-30LX
Type	Stereo and RGB-D	Monocular	Lidar
Resolution	1280 × 1024	2046 × 1086	0.25° (360°/1440 steps)
Frame Rate	60 fps	50 fps	40 fps (25 ms/scan)
Interface	USB 2.0	GigE	USB2.0

**Table 3:** Stereo Camera Used.

Parameter	Value
Product Model	3D-1MP02-V92
Sensor	OV9750
Lens Size	1/3 inch
Pixel size	3.75 um × 3.75 um
Highest effective pixel	2560 (H) × 960 (V)
Output image format	MJPEG
Signal to Noise Ratio	39 dB
Camera lens	Standard M9 lens FOV (D) 126 (H) 92 Degree
Sensitivity	3.7 V/lux-sec@550 nm
Shutter type	Electronic rolling shutter/Frame exposure
Interface type	USB 2.0 High Speed
Support free drive protocol	USB Video Class (UVC)
Support OTG protocol	USB 2.0 OTG
Automatic Exposure Control (AEC)	Support
Automatic White Balance (AEB)	Support
Automatic Gain Control (AGC)	Support
Support resolution	MJPEG: 340 × 240@64FPS 1280 × 480@64FPS 2560 × 720@64FPS 2560 × 960@64FPS
Power supply mode	MICRO USB
Supported systems	Win7 Win8 Linux 2.6 or above Android 4.0 or above

**Table 4:** The DFRobot MiniQ Encoder is Used.

Parameter	Value
Working Voltage	3.3 V or 5 V
Working Current	<14 mA @5 V
Pulse Output	12 per revolution
Compatibility	2 mm × 19 mm (1.65 × 0.75″) wheel
Receiver Sensitivity	Adjustable

**Table 5:** Analysis results of ORB-SLAM2 (ORB+BRIEF).

Error Analysis	Stereo Camera	Monocular Camera
RMSE	0.2323 m	3.5948 m
Mean	0.1940 m	3.4407 m
Median	0.1875 m	3.7131 m
Standard Deviation	0.1277 m	1.0414 m
Minimum Error	0.0068 m	0.9900 m
Maximum Error	0.7981 m	5.1786 m

**Table 6:** Analysis results of ORB-SLAM2 (ORB+BELID).

Error Analysis	Stereo Camera	Monocular Camera
RMSE	0.2329 m	3.6226 m
Mean	0.1957 m	3.4139 m
Median	0.1901 m	3.2597 m
Standard Deviation	0.1263 m	1.2118 m
Minimum Error	0.0136 m	2.0093 m
Maximum Error	0.8071 m	5.1181 m

**Table 7:** Increasing feature dimension from default 256 to 512 (ORB+BELID).

Error Analysis	Stereo Camera	Monocular Camera
RMSE	0.2321 m	4.0648 m
Mean	0.1935 m	3.8147 m
Median	0.1892 m	4.4489 m
Standard Deviation	0.1281 m	1.4041 m
Minimum Error	0.0063 m	1.1627 m
Maximum Error	0.8095 m	5.1062 m

**Table 8:** The default feature dimension is 256, but double the number of feature points (ORB+BELID).

Error Analysis	Stereo Camera	Monocular Camera
RMSE	0.2318 m	2.2692 m
Mean	0.1943 m	1.9656 m
Median	0.1859 m	2.2620 m
Standard Deviation	0.1264 m	1.1338 m
Minimum Error	0.0113 m	0.4421 m
Maximum Error	0.8070 m	3.3696 m

**Table 9:** Integration of front-end ORB+BELID with back-end loop closing weight optimization.

Error Analysis	Stereo Camera	Monocular Camera
RMSE	0.2316 m	2.9437 m
Mean	0.1933 m	2.6504 m
Median	0.1903 m	2.6205 m
Standard Deviation	0.1264 m	1.2810 m
Minimum Error	0.1275 m	1.0715 m
Maximum Error	0.8060 m	4.2889 m

## Data Availability

The EuRoC MAV dataset used in this study is publicly available at https://projects.asl.ethz.ch/datasets/doku.php?id=kmavvisualinertialdatasets (accessed on 18 August 2021) [41].
